# Supplementary material for: Ascorbate-Glutathione Cycle Genes Families in Euphorbiaceae: Characterization and Evolutionary Analysis
Source: Biology (Basel). 2022 Dec 22;12(1):19. doi: 10.3390/biology12010019 (PMC9855080; doi:10.3390/biology12010019)
Supplement: Supplementary file 1 [file biology-12-00019-s001.zip › biology-2055552-supplementary.pdf]

Figure S1

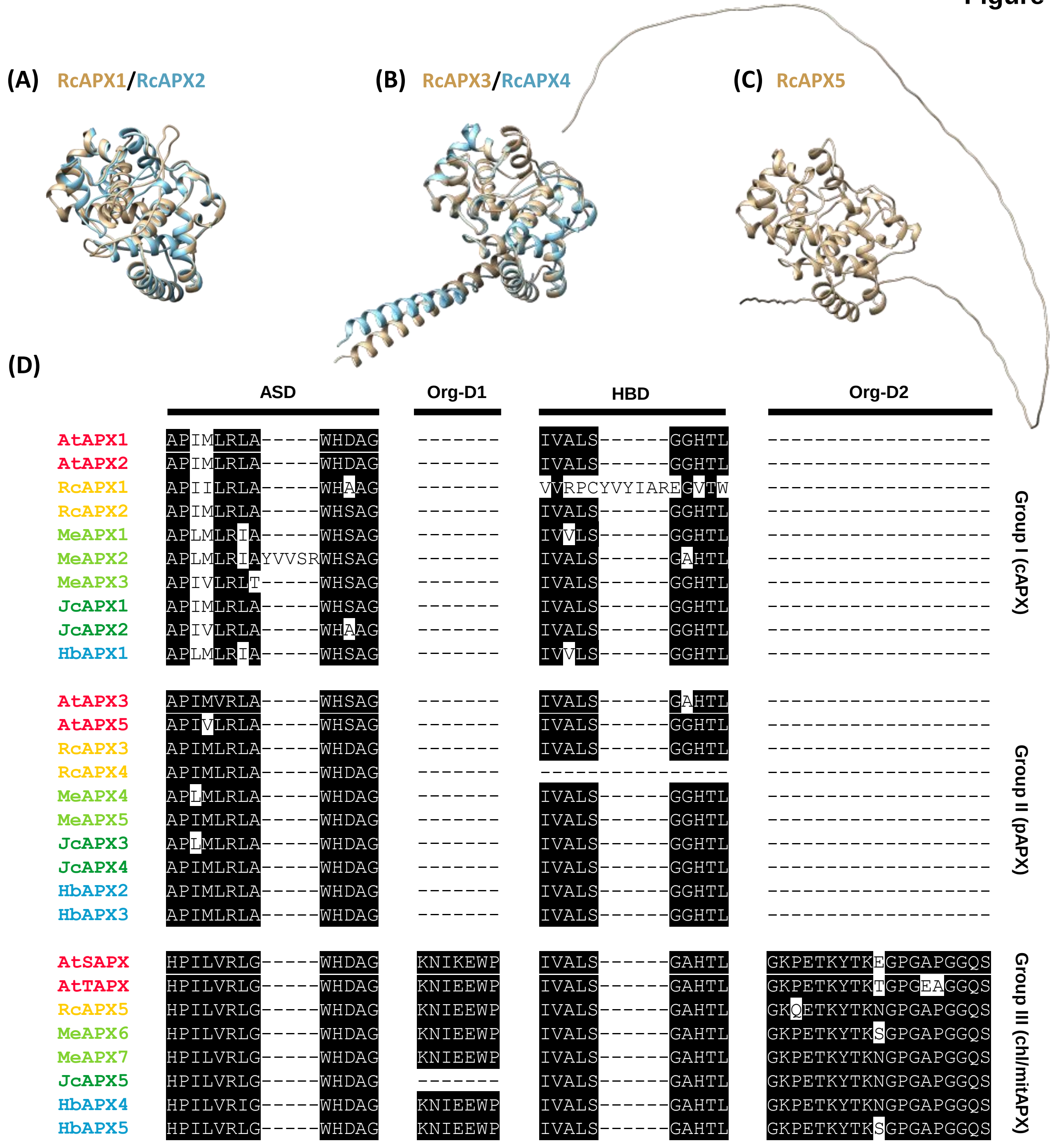

Figure S1. Structure and protein sequence analysis of APX family in *Ricinus communis* (Rc), *Manihot esculenta* (Me), *Jatropha curcas* (Jc), *Hevea brasiliensis* (Hb), *Arabidopsis thaliana* (At) and *Oryza sativa* (Os). Tertiary structure of RcAPX1 and RcAPX2 (cAPX) **(A)**, RcAPX3 and RcAPX4 (pAPX) **(B)** and RcAPX5 (chl/mitAPX) **(C)**. **(D)** Multiple sequence alignments of APX protein sequences. The black bars represent the active site domain (ASD), organelar signature domain 1 (Org-D1), heme-binding domain (HBD), and organelar signature domain 2 (Org-D2) present in all phylogenetic groups.

Figure S2

Motif	Logo	E-value	Sites	Width
1	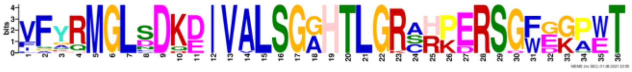	9.5e-409	17	36
2	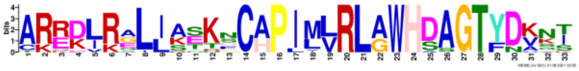	1.6e-370	19	33
3	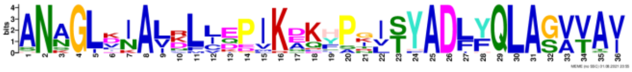	1.9e-345	19	36
4	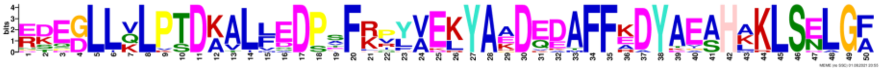	7.4e-488	18	50
5	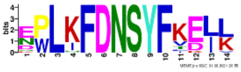	2.0e-148	18	14
6	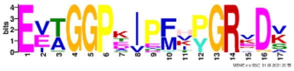	7.1e-139	18	17
7	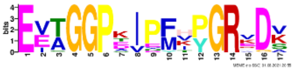	1.7e-112	19	14
8	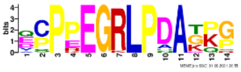	2.9e-097	17	14
9	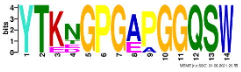	1.6e-043	7	14
10	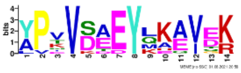	6.7e-034	11	14
11	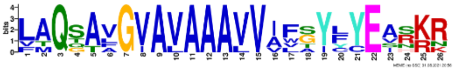	7.0e-030	6	26
12	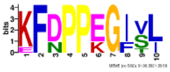	5.6e-012	7	10
13	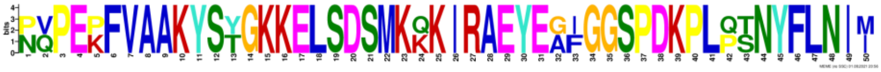	1.9e-007	2	50
14	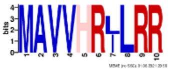	4.7e+001	2	10
15	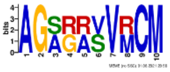	1.6e+003	2	10

Figure S2. Sequence logos for the conserved motifs of APX family from *Ricinus communis*, *Arabidopsis thaliana* and *Oryza sativa*. The logos were identified by MEME software. The character and size of each logo represent the proportion of an amino acid at the specific site. The statistical analysis indicating the probability of obtaining the same alignment score in a random database of the same size and the same amino acid composition is indicated, as well as the frequency of the motifs in the set of proteins analyzed (sites) and the size of the motif (width).

Figure S3

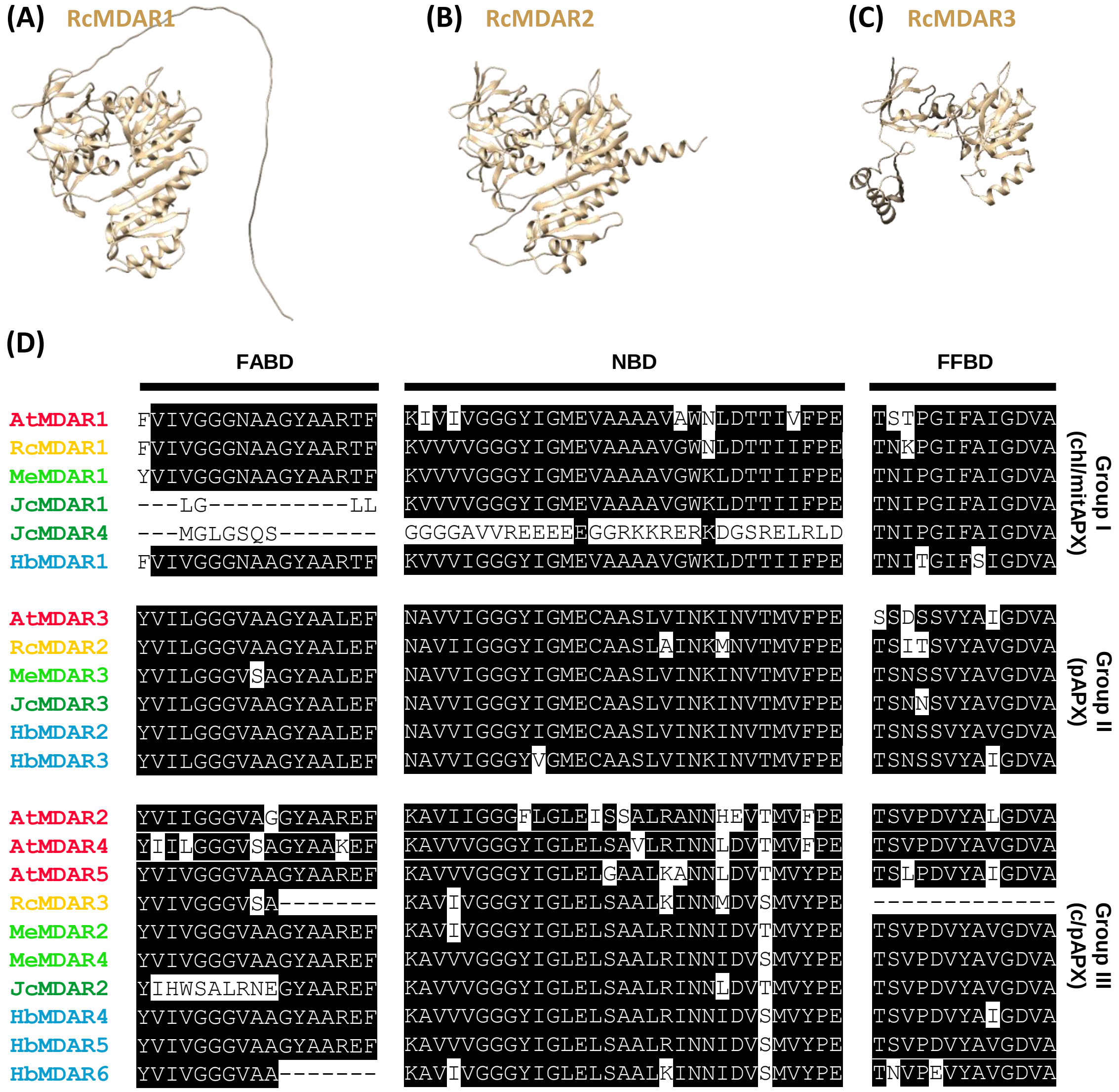

Figure S3. Structure and protein sequence analysis of MDAR family in *Ricinus communis* (Rc), *Manihot esculenta* (Me), *Jatropha curcas* (Jc), *Hevea brasiliensis* (Hb), *Arabidopsis thaliana* (At) and *Oryza sativa* (Os). Tertiary structure of RcMDAR1 (chl/mitMDAR) **(A)**, RcMDAR2 (pMDAR) **(B)** and RcMDAR3 (c/pMDAR) **(C)**. **(D)** Multiple sequence alignments of MDAR protein sequences. The black bars represent the FAD ADP-binding domain (FABD), NAD(P)H-binding domain (NBD), and FAD flavin-binding domain (FFBD), present in all phylogenetic groups.

Motif	Logo	E-value	Sites	Width
1	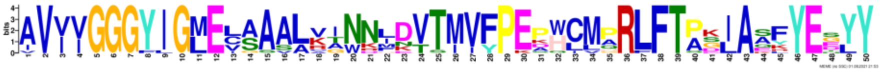	6.6e-344	13	50
2	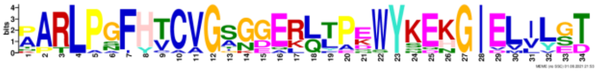	3.4e-219	13	34
3	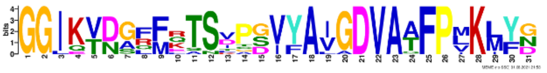	8.6e-150	12	31
4	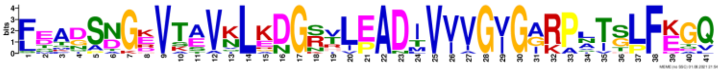	2.0e-196	13	41
5	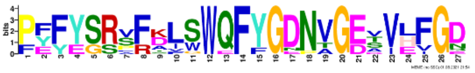	6.6e-419	12	27
6	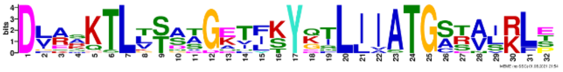	2.3e-231	13	32
7	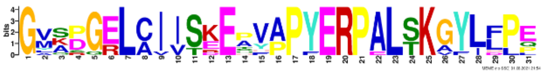	2.62e-090	11	31
8	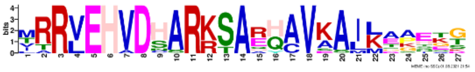	2.1e-086	12	27
9	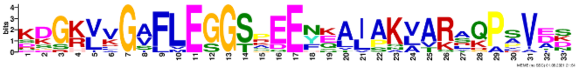	1.1e-073	13	33
10	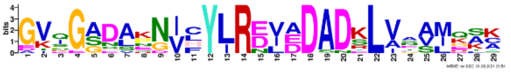	3.0e-047	13	29
11	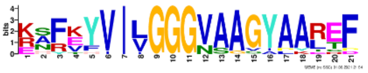	1.0e-039	13	21
12	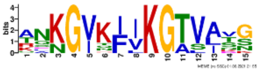	5.5e-026	12	15
13	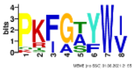	4.2e-024	13	8
14	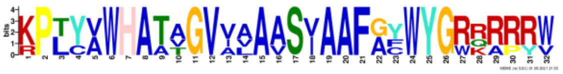	5.8e-018	4	32
15	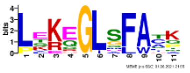	5.5e-013	12	11

Figure S4. Sequence logos for the conserved motifs of MDAR from *Ricinus communis*, *Arabidopsis thaliana* and *Oryza sativa*. The logos were identified by MEME software. The character and size of each logo represent the proportion of an amino acid at the specific site. The statistical analysis indicating the probability of obtaining the same alignment score in a random database of the same size and the same amino acid composition is indicated, as well as the frequency of the motifs in the set of proteins analyzed (sites) and the size of the motif (width).

Figure S5

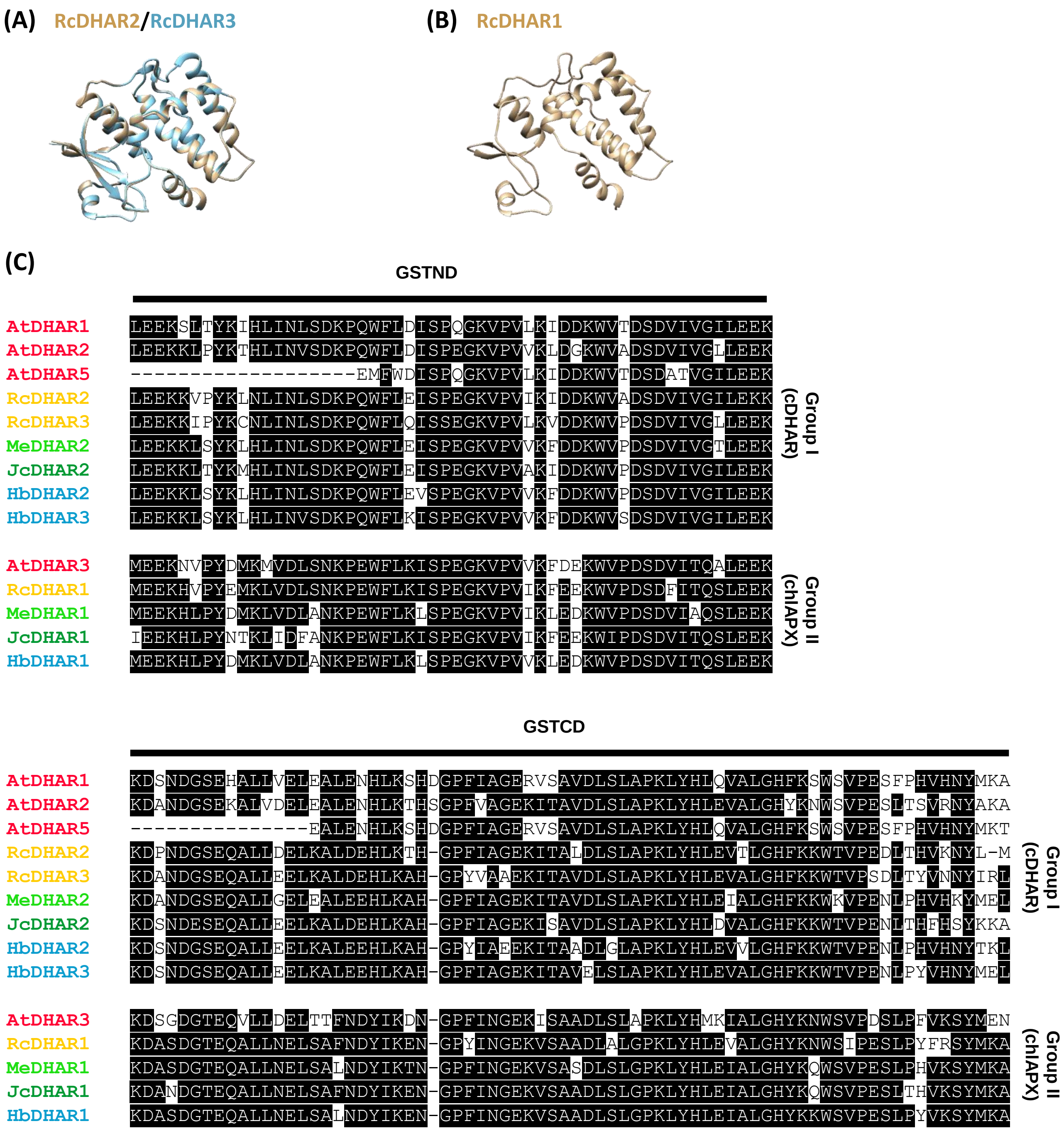

Figure S5. Structure and protein sequence analysis of DHAR family in *Ricinus communis* (Rc), *Manihot esculenta* (Me), *Jatropha curcas* (Jc), *Hevea brasiliensis* (Hb), *Arabidopsis thaliana* (At) and *Oryza sativa* (Os). Tertiary structure of RcDHAR2 and RcDHAR3 (cDHAR) **(A)** and RcDHAR1 (chlDHAR) **(B)**. **(C)** Multiple sequence alignments of DHAR protein sequences. The black bars represent the GST N-terminal domain (GSTND) and GST C-terminal domain (GSTCD), present in all phylogenetic groups.

Figure S6

Motif	Logo	E-value	Sites	Width
1	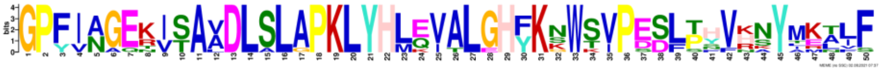	9.3e-211	9	50
2	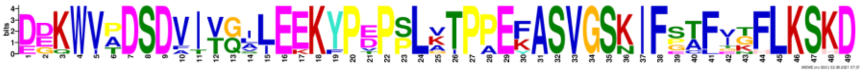	2.1e-202	9	49
3	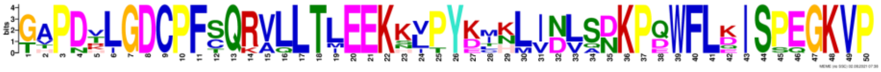	3.1e-156	7	50
4	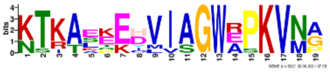	1.2e-058	9	19
5	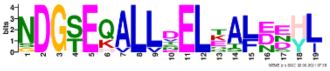	4.7e-042	8	19
6	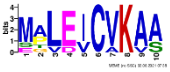	4.5e-16	7	10
7	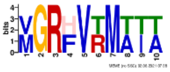	4.5e+001	2	10
8	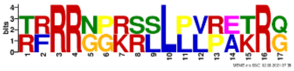	6.6e+002	2	17
9	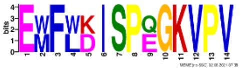	9.3e+002	2	14
10	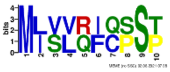	3.7e+003	2	10
11	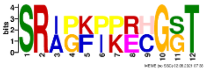	5.9e+003	2	12
12	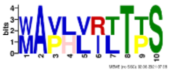	9.0e+004	2	10
13	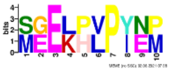	3.8e+005	2	10
14	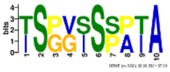	1.6e+006	2	10
15	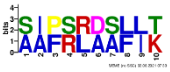	2.7e+006	2	10

Figure S6. Sequence logos for the conserved motifs of DHAR from *Ricinus communis*, *Arabidopsis thaliana* and *Oryza sativa*. The logos were identified by MEME software. The character and size of each logo represent the proportion of an amino acid at the specific site. The statistical analysis indicating the probability of obtaining the same alignment score in a random database of the same size and the same amino acid composition is indicated, as well as the frequency of the motifs in the set of proteins analyzed (sites) and the size of the motif (width).

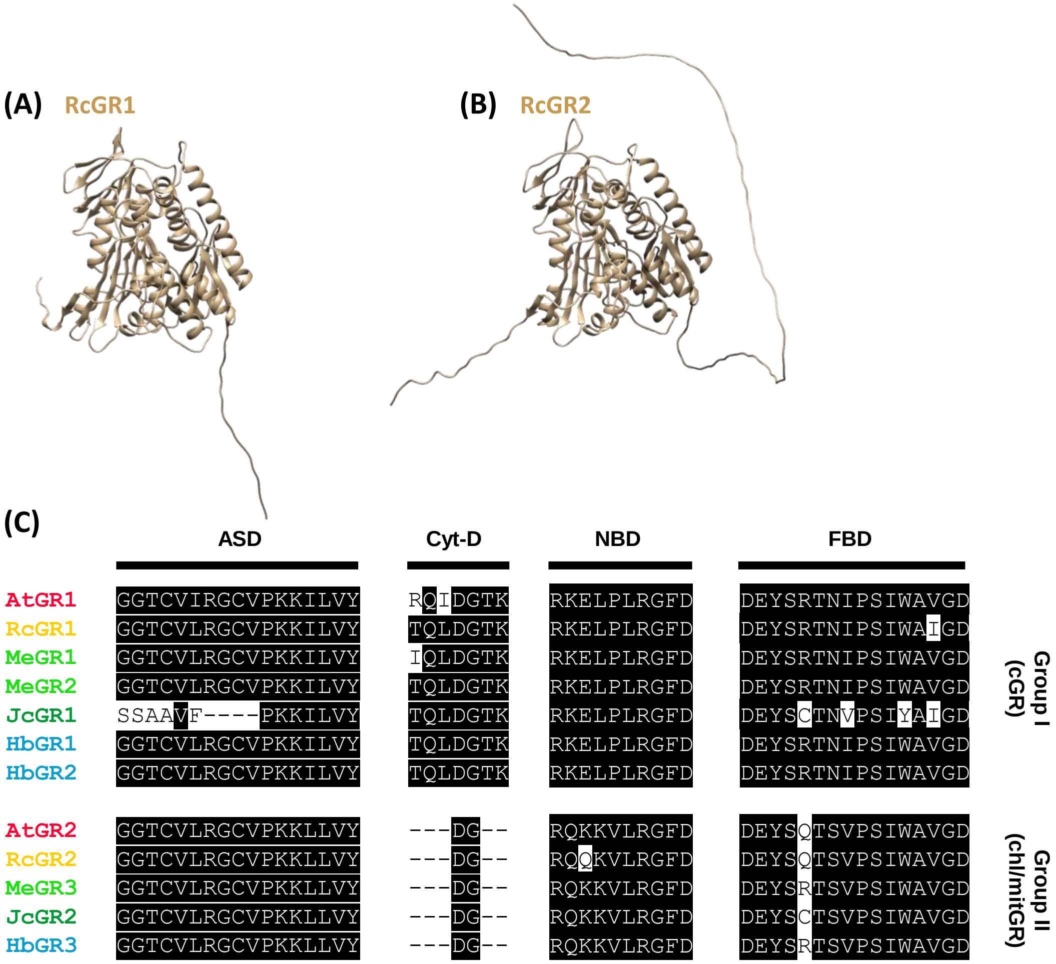

Figure S7. Structure and protein sequence analysis of GR family in *Ricinus communis* (Rc), *Manihot esculenta* (Me), *Jatropha curcas* (Jc), *Hevea brasiliensis* (Hb), *Arabidopsis thaliana* (At) and *Oryza sativa* (Os). Tertiary structure of RcGR1 (cGR) **(A)** and RcGR2 (chl/mitGR) **(B)**. **(C)** Multiple sequence alignments of GR protein sequences. The black bars represent the FAD-binding domain (FBD), the active site domain (ASD), cytosolic signature domain (Cyt-D), and NAD(P)H-binding domain (NBD), present in all phylogenetic groups.

Motif	Logo	E-value	Sites	Width
1	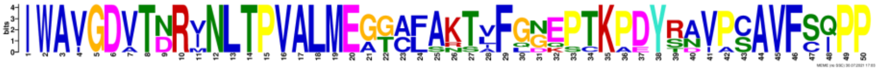	7.5e-171	7	50
2	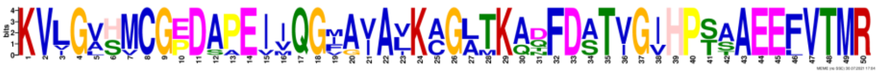	3.8e-163	7	50
3	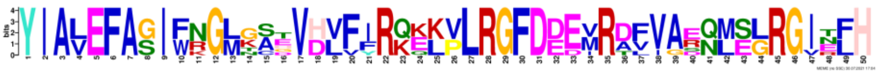	1.6e-143	7	50
4	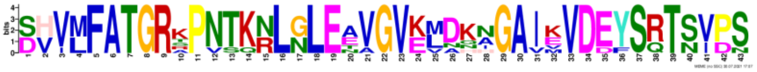	3.3e-117	7	43
5	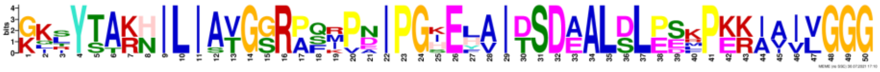	6.6e-419	7	50
6	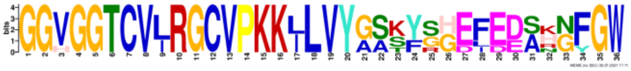	2.3e-231	6	36
7	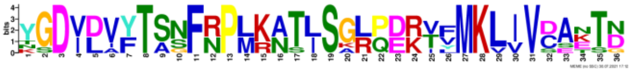	2.62e-090	7	36
8	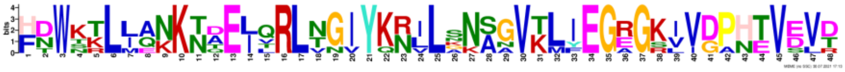	2.1e-086	6	48
9	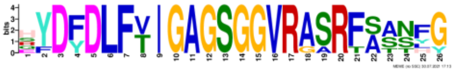	1.1e-073	6	26
10	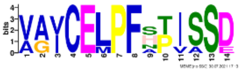	3.0e-047	6	14
11	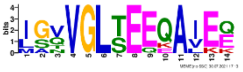	1.0e-039	7	14
12	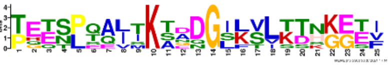	5.5e-026	7	25
13	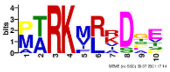	4.2e-024	7	10
14	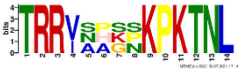	5.8e-018	3	14
15	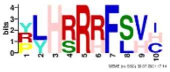	5.5e-013	3	10

Figure S8. Sequence logos for the conserved motifs of GR from *Ricinus communis*, *Arabidopsis thaliana* and *Oryza sativa*. The logos were identified by MEME software. The character and size of each logo represent the proportion of an amino acid at the specific site. The statistical analysis indicating the probability of obtaining the same alignment score in a random database of the same size and the same amino acid composition is indicated, as well as the frequency of the motifs in the set of proteins analyzed (sites) and the size of the motif (width).

Figure S9

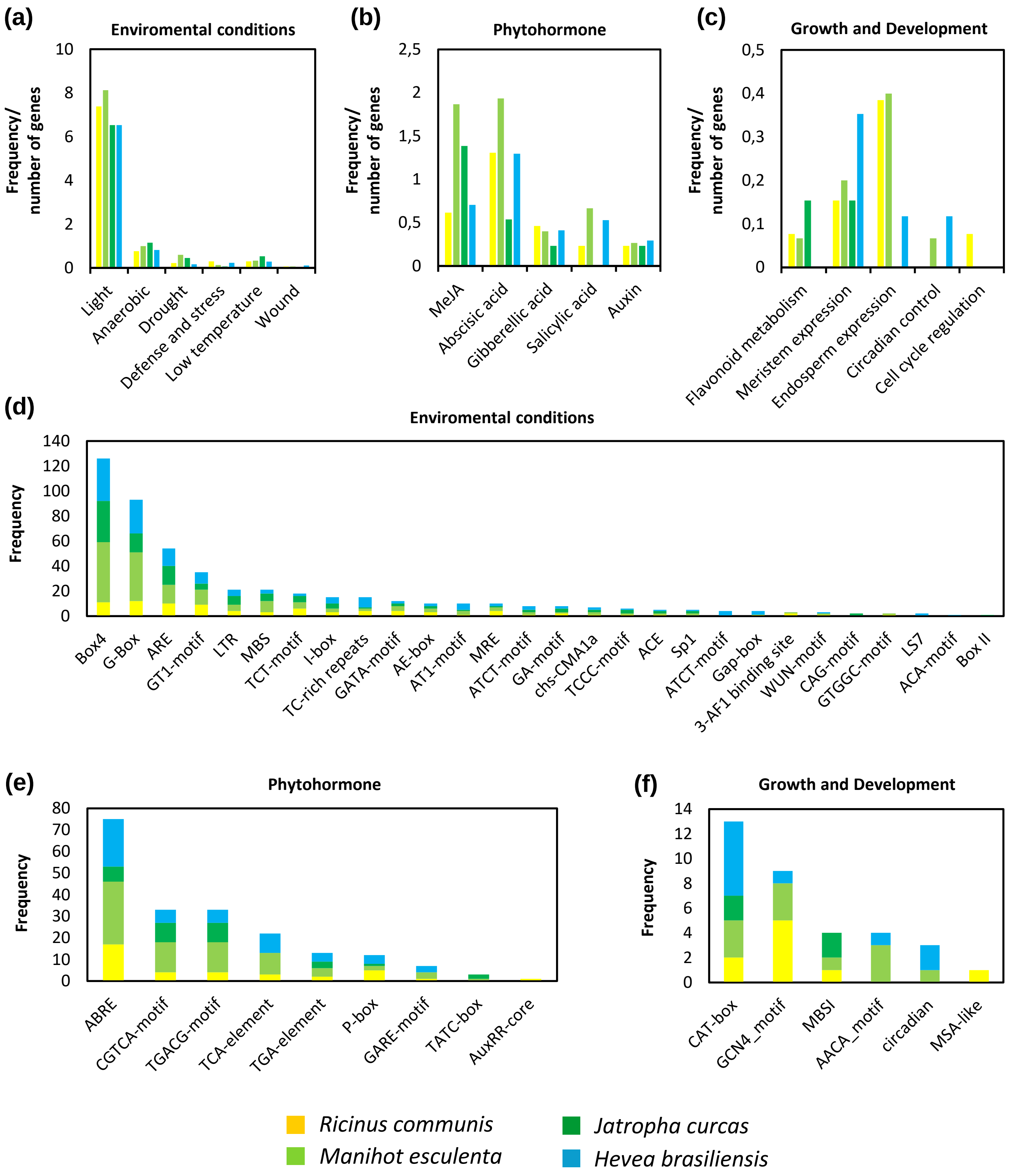
(a)
Enviromental conditions
Frequency/ number of genes
10
8
6
4
2
0
Light
Anaerobic
Drought
Defense and stress
Low temperature
Wound
(b)
Phytohormone
Frequency/ number of genes
2,5
2
1,5
1
0,5
0
MeJA
Abscisic acid
Gibberellic acid
Salicylic acid
Auxin
(c)
Growth and Development
Frequency/ number of genes
0,5
0,4
0,3
0,2
0,1
0
Flavonoid metabolism
Meristem expression
Endosperm expression
Circadian control
Cell cycle regulation
(d)
Enviromental conditions
Frequency
140
120
100
80
60
40
20
0
Box4
G-Box
ARE
GT1-motif
LTR
MBS
TCT-motif
I-box
TC-rich repeats
GATA-motif
AE-box
AT1-motif
MRE
ATCT-motif
GA-motif
chs-CMA1a
TCCC-motif
ACE
Sp1
ATCT-motif
Gap-box
3-AF1 binding site
WUN-motif
CAG-motif
GTGGC-motif
LS7
ACA-motif
Box II
(e)
Phytohormone
Frequency
80
70
60
50
40
30
20
10
0
ABRE
CGTCA-motif
TGACG-motif
TCA-element
TGA-element
P-box
GARE-motif
TATC-box
AuxRR-core
(f)
Growth and Development
Frequency
14
12
10
8
6
4
2
0
CAT-box
GCN4_motif
MBSI
AACA_motif
circadian
MSA-like
Ricinus communis
Manihot esculenta
Jatropha curcas
Hevea brasiliensis

Figure S9. Cis-regulatory elements in the *APX*, *MDAR*, *DHAR* and *GR* promoter regions from *Ricinus communis*, *Manihot esculenta*, *Jatropha curcas* and *Hevea brasiliensis*. (A), (B) and **(C)** The total number of cis-regulatory elements per number of genes involved in environmental conditions, phytohormones, and growth and development, respectively. Number of several cis-elements from each category: **(D)** environmental conditions, **(E)** phytohormones responsive, and **(F)** plant growth and development responsive. Diverse colors signify different species as indicated in the legend.

Figure S10

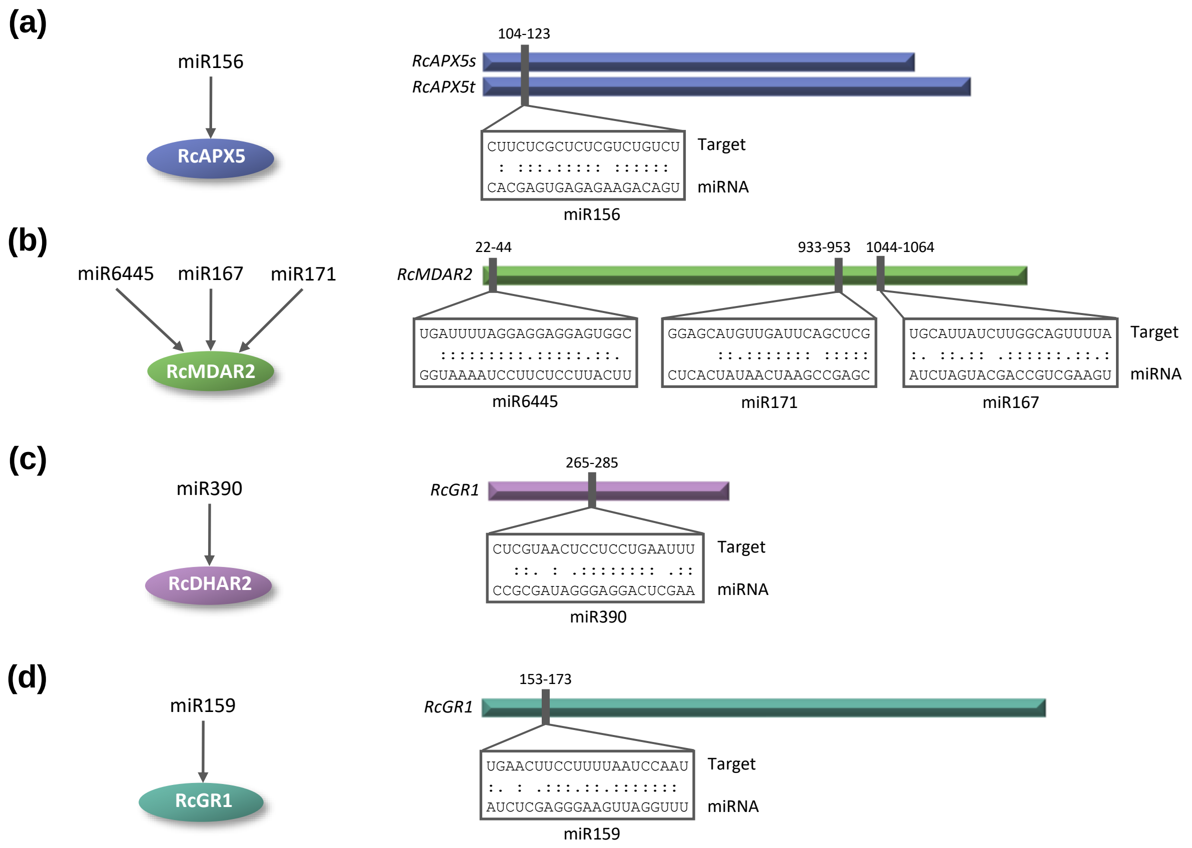

Figure S10. miRNA targeting *RcAPX*, *RcMDAR*, *RcDHAR*, and *RcGR* genes. The Graphic illustration indicates the *RcAPX5* gene is targeted by miRNA miR156 **(A)**, *RcMDAR2* gene is targeted by miRNAs miR6445, miR167 and miR171 **(B)**, *RcDHAR2* gene is targeted by miRNA miR390 **(C)**, and *RcGR1* gene is targeted by miRNA miR159 **(D)**. The grey thick bar indicates the location of miRNAs on each gene sequence. The RNA sequence of each complementary site and the predicted miRNA sequence are shown in the boxes. The complete dataset of predicted miRNAs is shown in Supplementary Table 1.

Table S1. APX, MDAR, DHAR and GR sequences from rice and arabidopsis used as bait to BLASTp analysis.

Arabidopsis thaliana TAIR10		*Oryza sativa* v7_JGI	
Name	Locus	Name	Locus
AtAPX1	*AT1G07890*	*OsAPX1*	*LOC_Os03g17690*
AtAPX2	*AT3G09640*	*OsAPX2*	*LOC_Os07g49400*
AtAPX3	*AT4G35000*	*OsAPX3*	*LOC_Os04g14680*
AtAPX5	*AT4G35970*	*OsAPX4*	*LOC_Os08g43560*
AtSAPX	*AT4G08390*	*OsAPX5*	*LOC_Os12g07830*
AtTAPX	*AT1G77490*	*OsAPX6*	*LOC_Os12g07820*
		OsAPX7	*LOC_Os04g35520*
		OsAPX8	*LOC_Os02g34810*
AtMDAR1	*AT1G63940*	*OsMDAR1*	*LOC_Os02g47790*
AtMDAR2	*AT3G09940*	*OsMDAR2*	*LOC_Os02g47800*
AtMDAR3	*AT3G27820*	*OsMDAR3*	*LOC_Os09g39380*
AtMDAR4	*AT3G52880*	*OsMDAR4*	*LOC_Os08g44340*
AtMDAR5	*AT5G03630*	*OsMDAR5*	*LOC_Os08g05570*
AtDHAR1	*AT1G19570*	*OsDHAR1*	*LOC_Os05g02530*
AtDHAR2	*AT1G75270*	*OsDHAR2*	*LOC_Os06g12630*
AtDHAR3	*AT5G16710*		
AtDHAR4	*AT5G36270*		
AtDHAR5	*AT1G19550*		
AtGR1	*AT3G24170*	*OsGR1*	*LOC_Os02g56850*
AtGR2	*AT3G54660*	*OsGR2*	*LOC_Os03g06740*
		OsGR3	*LOC_Os10g28000*

Table S2. Sequences of primers used in RT-qPCR experiments.

		Ricinus communis
APX family		
RcAPX1	F	5’GAAGCTCTCAGAACTTGGATT3’
	R	5’CACATGCTGCACTGAGCCT3’
RcAPX2	F	5’CTCGCTTGGGTTTTTCGCTT3’
	R	5’CAAGCCCATTATTAGCACCA3’
RcAPX3	F	5’AGACCCTGAGTTTCGTCCTT3’
	R	5’GCCACTAATTCATTTGGGACA3’
RcAPX4	F	5’GTTGAACGATATGCAGGGGA3’
	R	5’GAAGCTGTTTACTGCAAGAATT3’
RcAPX5-s	F	5’AGGGATGAAGACCTGCTTGT3’
	R	5’CTTGTTTCTTAATCCTTCCCA3’
RcAPX5-t	F	5’AGGGATGAAGACCTGCTTGT3’
	R	5’CAAGATACTTGTTTCTTAATCCT3’
MDAR family		
RcMDAR1	F	5’CTTCAGTTGAGGAGGCGCT3’
	R	5’AAATTGGCCGTGTAATTCTCAA3’
RcMDAR2	F	5’CAGTAGCTGCGTTCGGGTA3’
	R	5’GCAACTAAAGCACGATTATCTT3’
RcMDAR3	F	5’CAGTTTGGATCAGCTGACAAA3’
	R	5’GCAGAGGAACTTCGCTTTATA3’
DHAR family		
RcDHAR1	F	5’AACACGGGCTCTCCCAGAA3’
	R	5’TAAAGTGGCAGTGGAGAAACA3’
RcDHAR2	F	5’AAGGCTTCAAAGGAACATATG3’
	R	5’AACACATAACCTAGAACTTGCT3’
RcDHAR3	F	5’GGAACATGTGATTGCTGGCT3’
	R	5’GTTCACAAAACTCATAACTTAGA3’
GR Family		
RcGR1	F	5’TCGCTCACCAGGCGTGTTA3’
	R	5’CTTTGGAATCTCTCGCTGAT3’
RcGR2	F	5’GAACGACAGATCATGAGGTTA3’
	R	5’CAACCATAAAGGAGTTGCTGT3’
Reference genes		
RcADP	F	5’GAGAGATGCTGTGCTGCTTG3’
	R	5’TGAAGGCCGAGCTTATCAGT3’
RcEF1B	F	5’GCTCGAGGAAGCAGTTAGGA3’
	R	5’TTCCATATCCAACTGGCACA3’
RcPP2A	F	5’CCACAGGTTCTGGAGATGGT3’
	R	5’GAGCCCATTACAGGAGCAAG3’

Table S3. Physicochemical parameters and subcellular predictions from APX in *Ricinus communis, Manihot esculenta, Jatropha curcas, Hevea brasiliensis*. Gene name: proposed nomenclature; locus ID; phylogenetic group; size in aminoacid, Mass - molecular weight; pI - isoelectric point; GRAVY - grand average of hydropathy; Instability Index.

Specie Name	Name	Gene ID	Group	Ptn Length	MW (Da)	PI	GRAVY	Instability Index
Ricinus communis	RcAPX1	29805.m001492	I	259	28775.91	5.56	-0.422	35.47 (stable)
	RcAPX2	29781.m000013	I	223	24549.07	6.71	-0.431	33.50 (stable)
	RcAPX3	29602.m000217	II	288	32059.56	7.13	-0.422	41.63 (unstable)
	RcAPX4	29656.m000477	II	235	25878.50	8.22	-0.338	34.61 (stable)
	RcAPX5	29648.m002024	III	379	40843.15	7.69	-0.380	49.33 (unstable)
Manihot esculenta	MeAPX1	Manes.04G026800	I	250	27669.34	5.31	-0.406	41.10 (unstable)
	MeAPX2	Manes.11G139700	I	255	28138.95	6.09	-0.410	39.59 (stable)
	MeAPX3	Manes.08G002700	I	250	27724.44	6.00	-0.462	36.78 (stable)
	MeAPX4	Manes.01G147300	II	282	31484.91	7.77	-0.446	40.59 (unstable)
	MeAPX5	Manes.11G094500	II	288	31615.01	7.06	-0.302	34.17 (stable)
	MeAPX6	Manes.18G078800	III	375	40663.86	7.68	-0.401	56.42 (unstable)
	MeAPX7	Manes.02G165100	III	428	46435.72	7.65	-0.306	48.20 unstable
Jatropha curcas	JcAPX1	Jcr4S00918.60	I	250	27500.15	5.41	-0.396	33.52 (stable)
	JcAPX2	Jcr4S00717.110	I	211	23692.15	9.36	-0.500	45.99 (unstable)
	JcAPX3	Jcr4S06417.20	II	286	31843.42	8.82	-0.449	40.82 (unstable)
	JcAPX4	Jcr4S00147.10	II	357	39269.10	7.71	-0.119	32.65 (stable)
	JcAPX5	Jcr4S00512.40	III	397	42910.88	8.21	-0.235	50.71 (unstable)
Hevea brasiliensis	HbAPX1	HBR0213G001	I	250	27429.11	5.78	-0.362	37.45 (stable)
	HbAPX2	HBR2353G021	II	282	31031.40	8.22	-0.385	37.68 (stable)
	HbAPX3	HBR2728G031	II	285	31632.30	8.25	-0.328	37.05 (stable)
	HbAPX4	HBR2837G001	III	375	40896.36	8.47	-0.387	56.22 (unstable)
	HbAPX5	HBR2415G005	III	413	45263.51	8.19	-0.292	49.37 (unstable)

Table S4. Physicochemical parameters and subcellular predictions from MDAR in *Ricinus communis*, *Manihot esculenta*, *Jatropha curcas*, *Hevea brasiliensis*. Gene name: proposed nomenclature; locus ID; phylogenetic group; size in aminoacid, Mass - molecular weight; pI - isoelectric point; GRAVY - grand average of hydropathy; Instability Index.

Specie Name	Name	Gene ID	Group	Ptn Length	MW (Da)	PI	GRAVY	Instability Index
Ricinus communis	RcMDAR1	30190.m011270	I	493	53885.56	8.42	-0.147	31.18 (stable)
	RcMDAR2	29917.m001987	II	478	51871.39	8.30	-0.008	38.05 (stable)
	RcMDAR3	27461.m000101	III	312	33442.82	8.69	0.066	22.62 (stable)
Manihot esculenta	MeMDAR1	Manes.01G057400	I	497	54369.15	8.35	-0.135	31.77 (stable)
	MeMDAR2	Manes.08G023066	III	434	47179.82	5.60	-0.120	31.05 (stable)
	MeMDAR3	Manes.15G149700	II	478	51892.50	8.94	-0.032	38.67 (stable)
	MeMDAR4	Manes.09G058561	III	434	47117.88	5.73	-0.084	26.08 stable
Jatropha curcas	JcMDAR1	Jcr4S00535.90	I	594	65863.58	6.82	-0.277	33.51 (stable)
	JcMDAR2	Jcr4S10295.10	III	458	50153.45	6.00	-0.078	29.41 (stable)
	JcMDAR3	Jcr4S00820.50	II	451	48957.00	8.39	-0.004	38.99 (stable)
	JcMDAR4	Jcr4S26159.10	I	265	29316.98	8.77	-0.553	54.11 (unstable)
Hevea brasiliensis	HbMDAR1	HBR2013G001	I	453	49682.70	8.54	-0.138	30.15 (stable)
	HbMDAR2	HBR2708G019	II	480	52389.97	8.56	-0.019	37.52 (stable)
	HbMDAR3	HBR1414G024	II	478	52193.91	8.50	0.032	39.23 (stable)
	HbMDAR4	HBR2926G097	III	434	47124.90	5.90	-0.086	27.43 (stable)
	HbMDAR5	HBR1888G014	III	434	47109.89	6.12	-0.082	27.19 (stable)
	HbMDAR6	HBR1624G001	III	410	44543.97	5.87	-0.063	28.09 (stable)

Table S5. Physicochemical parameters and subcellular predictions from DHAR in *Ricinus communis*, *Manihot esculenta*, *Jatropha curcas*, *Hevea brasiliensis*. Gene name: proposed nomenclature; locus ID; phylogenetic group; size in aminoacid, Mass - molecular weight; pI - isoelectric point; GRAVY - grand average of hydropathy; Instability Index.

Specie Name	Name	Gene ID	Group	Ptn Length	MW (Da)	PI	GRAVY	Instability Index
Ricinus communis	RcDHAR1	28524.m000012	II	183	20684.77	5.32	-0.323	36.80 (stable)
	RcDHAR2	29939.m000529	I	211	23653.46	5.89	-0.199	25.74 (stable)
	RcDHAR3	29939.m000525	I	212	23561.25	5.78	-0.107	29.44 (stable)
Manihot esculenta	MeDHAR1	Manes.01G117300	II	272	30006.62	8.78	-0.187	45.21 (unstable)
	MeDHAR2	Manes.10G037200	I	212	23709.50	5.92	-0.174	37.20 (stable)
Jatropha curcas	JcDHAR1	Jcr4S07524.30	II	219	24377.10	5.88	-0.215	42.63 (unstable)
	JcDHAR2	Jcr4S05806.20	I	199	22201.64	5.75	-0.158	36.44 (stable)
Hevea brasiliensis	HbDHAR1	HBR1255G003	II	273	30267.01	8.48	-0.143	45.92 (unstable)
	HbDHAR2	HBR0154G034	I	212	23783.54	6.08	-0.215	37.97 (stable)
	HbDHAR3	HBR2353G016	I	212	23815.53	5.69	-0.163	36.63 (stable)

Table S6. Physicochemical parameters and subcellular predictions from GR in *Ricinus communis, Manihot esculenta, Jatropha curcas, Hevea brasiliensis*. Gene name: proposed nomenclature; locus ID; phylogenetic group; size in aminoacid, Mass - molecular weight; pI - isoelectric point; GRAVY - grand average of hydropathy; Instability Index.

Specie Name	Name	Gene ID	Group	Ptn Length	MW (Da)	PI	GRAVY	Instability Index
Ricinus communis	RcGR1	29904.m002966	I	496	53583.34	5.74	-0.112	26.81 (stable)
	RcGR2	29883.m001999	II	560	60050.43	7.20	-0.100	36.28 (stable)
Manihot esculenta	MeGR1	Manes.03G149200	I	496	53780.53	5.86	-0.131	30.09 (stable)
	MeGR2	Manes.15G051800	I	495	54344.30	6.20	-0.098	25.65 stable
	MeGR3	Manes.05G084700	II	559	60410.96	7.22	-0.143	40.23 (unstable)
Jatropha curcas	JcGR1	Jcr4S00410.70	I	488	52709.27	5.63	-0.097	31.93 (stable)
	JcGR2	Jcr4S02009.80	II	555	59701.36	8.05	-0.099	35.65 (stable)
Hevea brasiliensis	HbGR1	HBR1198G047	I	496	53573.17	5.79	-0.138	25.48 (stable)
	HbGR2	HBR0276G003	I	496	53684.44	6.18	-0.141	28.34 (stable)
	HbGR3	HBR0994G100	II	557	60408.12	7.20	-0.112	39.95 (stable)

Table S7. Conserved miRNAs targeting AsA-GSH genes in castor bean. miRNAn ame; target of miRNA; expectation (Exp.); UPE$; proposed alignment; inhibition type; multiplicity (Mult.).

miRNA	Target_Acc.	Exp.	UPE$	alignment	Inhibition	Mult.
miR156a	RcAPX5s	4	-1	: :::.::::: ::::::	Cleavage	1
miR156a	RcAPX5t	4	-1	: :::.::::: ::::::	Cleavage	1
miR156b	RcAPX5t	4,5	-1	: ::: ::::: ::::::	Cleavage	1
miR156b	RcAPX5s	4,5	-1	: ::: ::::: ::::::	Cleavage	1
miR156f	RcAPX5t	4	-1	: :::.::::: ::::::	Cleavage	1
miR156f	RcAPX5s	4	-1	: :::.::::: ::::::	Cleavage	1
miR156h	RcAPX5t	4	-1	: :::.::::: ::::::	Cleavage	1
miR156h	RcAPX5s	4	-1	: :::.::::: ::::::	Cleavage	1
miR156i	RcAPX5t	4	-1	: :::.::::: ::::::	Cleavage	1
miR156i	RcAPX5s	4	-1	: :::.::::: ::::::	Cleavage	1
miR159a	RcGR1	4,5	-1	:. : .:::.::.:::::::	Cleavage	1
miR167a	RcMDAR2	4,5	-1	::. ::.:: .::::::.::.:	Cleavage	1
miR167b	RcMDAR2	4,5	-1	:. ::.:: .::::::.::.:	Cleavage	1
miR167c	RcMDAR2	4	-1	:. ::.:: .::::::.::::	Cleavage	1
miR167d	RcMDAR2	4,5	-1	::.:: .::::::.::.:	Cleavage	1
miR167e	RcMDAR2	4,5	-1	::.:: .::::::.::.:	Cleavage	1
miR171e	RcMDAR2	5	-1	::.::::::: :::::	Cleavage	1
miR390a	RcDHAR2	5	-1	::. : .:::::::: .::	Cleavage	1
miR390b	RcDHAR2	5	-1	::. : .:::::::: .::	Cleavage	1
miR6445	RcMDAR2	2,5	-1	:::::::::.:::::.::.	Cleavage	1

Expectation: Expectation value is the penalty for the mismatches between mature small RNA and the target sequence. A higher value indicates less similarity (and possibility) between small RNA and the target candidate. The default penalty rule is set up by the scoring schema. Maximum expectation is the cutoff; any small RNA-target pair with an expectation less than the cutoff will be discarded in the final result. The recommended values are 3.0-5.0 depending on the scoring schema. UPE: The accessibility of the mRNA target site to small RNA has been identified as one of the important factors involved in target recognition because the secondary structure (stem etc.) around the target site will prevent small RNA (including miRNA and ta-siRNA, sic passim) and the mRNA target from having contact. The psRNATarget server employes RNAup to calculate target accessibility, which is represented by the energy required to open (unpair) the secondary structure around the target site (usually the complementary region with small RNA and up/downstream) on target mRNA(see figure below). Less energy means more possibility that small RNA is able to contact (and cleave) target mRNA. Multiplicity: number of target sites for each small RNA/target pair
